# Adeno-Associated Viral Vectors Serotype 8 for Cell-Specific Delivery of Therapeutic Genes in the Central Nervous System

**DOI:** 10.3389/fnana.2017.00002

**Published:** 2017-02-10

**Authors:** Diego Pignataro, Diego Sucunza, Lucia Vanrell, Esperanza Lopez-Franco, Iria G. Dopeso-Reyes, Africa Vales, Mirja Hommel, Alberto J. Rico, Jose L. Lanciego, Gloria Gonzalez-Aseguinolaza

**Affiliations:** ^1^Department of Gene Therapy and Regulation of Gene Expression, Center for Applied Medical ResearchPamplona, Spain; ^2^Department of Neurosciences, Center for Applied Medical ResearchPamplona, Spain; ^3^Centro de Investigación Biomédica en Red sobre Enfermedades NeurodegenerativasSpain; ^4^Instituto de Investigación Sanitaria de NavarraPamplona, Spain

**Keywords:** AAV, CNS, promoters, basal ganglia, Parkinson's disease, gene therapy

## Abstract

Adeno-associated viruses (AAVs) have become highly promising tools for research and clinical applications in the central nervous system (CNS). However, specific delivery of genes to the cell type of interest is essential for the success of gene therapy and therefore a correct selection of the promoter plays a very important role. Here, AAV8 vectors carrying enhanced green fluorescent protein (eGFP) as reporter gene under the transcriptional control of different CNS-specific promoters were used and compared with a strong ubiquitous promoter. Since one of the main limitations of AAV-mediated gene delivery lies in its restricted cloning capacity, we focused our work on small-sized promoters. We tested the transduction efficacy and specificity of each vector after stereotactic injection into the mouse striatum. Three glia-specific AAV vectors were generated using two truncated forms of the human promoter for glial fibrillar acidic protein (GFAP) as well as a truncated form of the murine GFAP promoter. All three vectors resulted in predominantly glial expression; however we also observed eGFP expression in other cell-types such as oligodendrocytes, but never in neurons. In addition, robust and neuron-specific eGFP expression was observed using the minimal promoters for the neural protein BM88 and the neuronal nicotinic receptor β2 (CHRNB2). In summary, we developed a set of AAV vectors designed for specific expression in cells of the CNS using minimal promoters to drive gene expression when the size of the therapeutic gene matters.

## Introduction

Longevity coincides with an increased prevalence in neurodegenerative disease and a concomitant increase in the burden on health systems around the world (Checkoway et al., 2011). The need for treatment options has fuelled research, with the field of gene therapy applied to central nervous system (CNS) pathologies being on the forefront. Despite having recently witnessed a number of major conceptual changes—such as gene delivery of specific transcription factors or micro-RNAs for *in vivo* reprogramming of different cells to neurons (Caiazzo et al., 2011; Niu et al., 2013, 2015; Colasante et al., 2015; Ghasemi-Kasman et al., 2015)—the more traditional approach of using viral vectors for the delivery of therapeutic genes still offers one of the most promising options (Terzi and Zachariou, 2008; Bartus et al., 2013; Kalia et al., 2015).

Although viral and non-viral vectors have been broadly used for CNS gene therapy, viral vectors, including adeno-associated viruses (AAVs) and lentiviruses (Blessing and Déglon, 2016), are generally significantly more efficient than non-viral vectors at delivering genes into the cells of interest (Nayerossadat et al., 2012). Cell-specificity can be directed by either intrinsic characteristics of the vector (Nayerossadat et al., 2012; Kantor et al., 2014; Maguire et al., 2014) or the specificity of the promoter that controls the expression of the transgene (Gray et al., 2011). AAVs have emerged as the most promising tool for gene transfer in the CNS (Klein et al., 2007; Aschauer et al., 2013; Bourdenx et al., 2014) as they are able to transduce dividing and non-dividing cells and induce stable, long-term gene expression in the absence of inflammation and/or toxicity. Since neurons are post-mitotic cells, the capability of AAV vectors to transduce non-dividing cells is of vital importance in the context of neurodegenerative disease gene therapy (Bartlett et al., 2008).

AAV serotype 8 (AAV8) in particular has been demonstrated to be one of the most effective vectors in some structures of the CNS, producing the highest rate of transgene transduction in the striatum compared with other serotypes, in the absence of neurotoxicity (Aschauer et al., 2013). Moreover, in a number of studies in different animal models it was observed that this serotype was actively transported along axons (Masamizu et al., 2010, 2011; Aschauer et al., 2013; Löw et al., 2013). Due to its small size (4.7 kb) one of its limitations is its cloning capacity, however, the use of minimal specific promoters facilitates the expression of larger genes or co-expression of more than one gene from the same vector. In pre-clinical and clinical studies the use of AAV as delivery vehicles was confirmed to result in robust and long-term gene expression (reviewed by Hocquemiller et al., 2016).

In the present work we describe the characterization of a series of astrocyte- and neuron-specific small promoters in the context of an AAV8 vector with the aim of using these vectors for future therapeutic applications in neurodegenerative disease including Parkinson‘s disease (Coune et al., 2012). Astrocytes were chosen as they are one of the most abundant cell types in the vertebrate CNS (Colombo and Farina, 2016) and contribute to the pathogenesis of neurodegenerative disorders—hence they may be an ideal cellular target for the delivery of therapeutic genes (Pekny and Nilsson, 2005). Because the anatomy of the striatum is affected in many neurodegenerative diseases, such as Parkinson's disease, we characterized the expression pattern and specificity of the different vectors by stereotaxic injection into the mouse striatum. Robust and specific neuronal transgene expression was achieved using neuron-specific promoters, while astrocyte-specific promoters drove expression in astrocytes and oligodendrocytes but not in neurons.

## Materials and methods

### Animals and stereotaxic AAV injection

Eighteen C57BL/6 male mice (6–8 weeks old) were purchased from Harlan Laboratories (Barcelona, Spain). Animal handling was conducted in accordance with the European Council Directive 2010/63/UE, as well as in agreement with the “Policy on the Use of Animals in Neuroscience Research” issued by the Society for Neuroscience. The experimental design was approved by the Ethical Committee for Animal Testing of the University of Navarra (protocol Ref: 102-16). Animal handling was conducted in accordance with the European Council Directive 2010/63/UE, as well as in agreement with the “Policy on the Use of Animals in Neuroscience Research” issued by the Society for Neuroscience. The experimental design was approved by the Ethical Committee for Animal Testing of the University of Navarra (protocol Ref: 102-16). Anesthesia was induced by intraperitoneal injection of ketamine (100 mg/kg) and xylazine (10 mg/kg). The coordinates for targeting the striatum were 0.5 mm rostral, 2 mm lateral and 3.5 mm ventral from the bregma (Paxinos et al., 2001). All animals received two pressure injections: one of 2 μl of PBS/5% sucrose containing AAV vector on the left side (4 × 10^9^ vp), and a second of vehicle alone on the right side of the striatum. Injections were performed using a Hamilton syringe driven by a syringe pump at a flow rate of 0.2 μl/min. Following the injection, the needle was left in place for 2 min prior to being slowly retracted to avoid vector leakage from the injection tract. After surgery, animals were kept under constant monitoring with *ad libitum* access to food and water.

### Cells

Human embryonic kidney fibroblast (HEK-293) cells, were purchased from the ATCC and were cultured in Dulbecco's modified Eagle's medium (DMEM) supplemented with 10% (v/v) heat-inactivated fetal bovine serum (FBS), penicillin (100 μg/ml), and streptomycin (100 U/ml) (all supplements were from Invitrogen, Pisley, Scotland, UK). Cells were maintained at 37°C in a humidified atmosphere of 5% CO_2_.

### Plasmids

**Figure 1:**
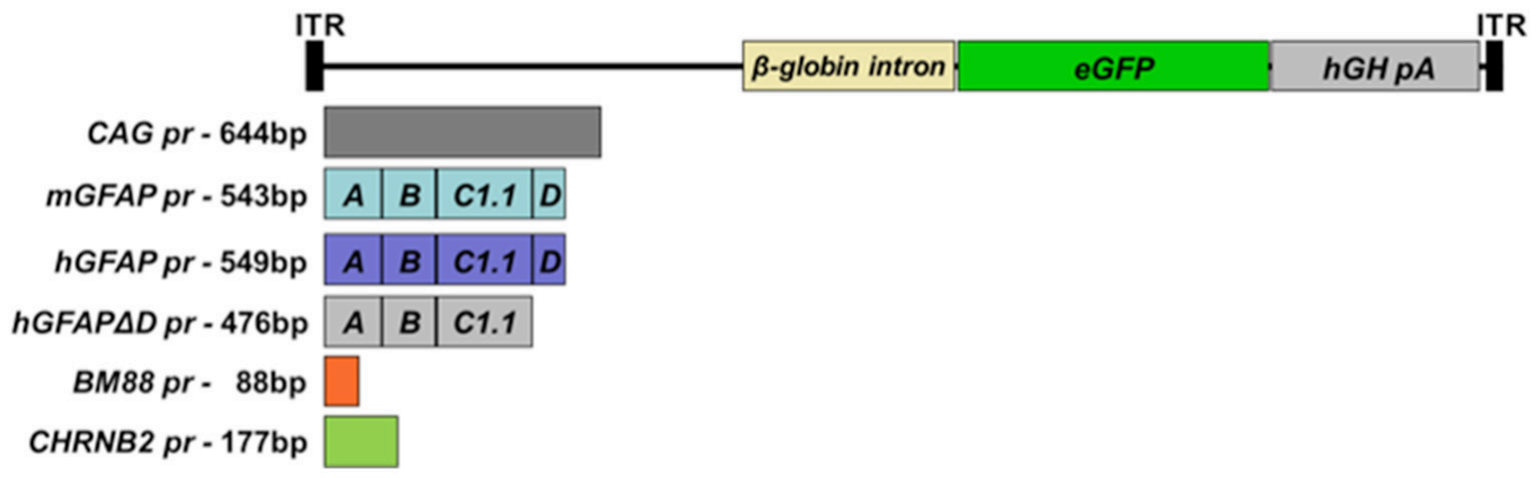
**Schematic representation of the genomic structures of the AAV vectors**. AAV vectors carrying reduced versions of the human or murine astrocyte-specific GFAP promoters, the minimal neuronal promoters BM88 or CHNRB2, which control the expression of the reporter gene enhanced GFP (eGFP). The expression cassettes also contain the ß-globin intron downstream of the promoter sequence and the human growth hormone polyadenylation signal sequence (hGH-polyA).

cDNA encoding enhanced green fluorescent protein (eGFP) was isolated from the vector pBSKII-CMV-EGFP and inserted into the multiple cloning site (MCS) of an rAAV2 plasmid, which contained AAV2 inverted terminal repeats (ITR), to obtain rAAV2-eGFP. Upstream of the eGFP coding sequence different promoters were inserted: a constitutive hybrid promoter composed of the CMV immediate-early enhancer fused to chicken ß-actin promoter (CAG pr; Niwa et al., 1991), two reduced versions of the human glial fibrillar acidic protein (GFAP) promoter (hGFAP pr, 587 bp, containing the A, B, C_1_, and D elements) (Lee et al., 2008) and hGFAPΔD (512 bp), in which the D sequence of was removed. This sequence was previously shown to play an important role in the functionality of the promoter (Besnard et al., 1991). Furthermore, using the structure of the human gfaABC_1_D promoter and the sequence of the murine GFAP promoter, a reduced version of the murine gfaABC_1_D promoter was constructed (581 bp, mGFAP pr). The proximal promoter of murine BM88 (88 bp; Papadodima et al., 2005) and the minimal promoter driving neuron-specific expression of the ß2 subunit of the nicotine acetylcholine receptor (CHNRB2 pr, 177 bp; Bessis et al., 1995), were used for neuron-specific targeting. Moreover, the human growth hormone (hGH) poly A signal and the ß-globin intron were cloned into the plasmid (Figure 1). Minipreps and maxipreps were prepared using commercial kits according to the manufacturer's instructions (Macherey-Nagel, Düren, Germany). In order to study the functionality of the constructs, HEK-293T cells were transfected with plasmid DNA using Lipofectamine 2000 reagent (Invitrogen, ThermoFisher Scientific, Waltham, MA, USA). Transfections were performed according to the manufacturer's protocols. 1–2 μg of plasmid was transfected, depending on the size of the culture plate used (6- or 12-wells). Expression was analyzed 24–48 h post-transfection (hpt) under a microscope equipped with epifluorescent illumination (Nikon Eclipse 800).

### Viral vector production

Recombinant single-stranded AAV8 vectors were purified from HEK-293T cells that had been co-transfected using linear polyethylenimine 25 kDa (Polysciences, Warrington, PA, USA) with two different plasmids: a plasmid containing ITR-flanked transgene constructs and a plasmid containing the adenoviral helper genes plus AAV2 rep and AAV8 cap (named pDP8.ape, Plasmid Factory, Bielefeld, Germany) as described (Durocher et al., 2002). Seventy-two hpt the supernatant was collected and treated with polyethylene glycol solution (PEG8000, 8% v/v final concentration) for 48–72 h at 4°C. Supernatant was then centrifuged at 3000 rpm for 15 min. Pellet containing particles from the supernatant was resuspended in lysis buffer and kept at −80°C. Cells containing AAV particles were collected and treated with lysis buffer (50 mM Tris-Cl, 150 mM NaCl, 2 mM MgCl_2_, 0.1% Triton X-100) and kept at −80°C. Three cycles of freezing and thawing were applied to both supernatant and cell lysate. Viral particles obtained from cell supernatant and lysate were purified by ultracentrifugation in an iodioxanol gradient according to the method of Zolotukhin et al. (1999). The viral batches were then concentrated further by passage through centricon tubes (YM-100; Millipore, Bedford, MA). All vector stocks were kept at −80°C until used.

AAV vector titers (viral particles (vp)/ml) were determined by quantitative PCR for viral genome copies extracted from DNAase-treated viral particles (High Pure Viral Nucleic Acid Kit, Roche). The primers used in the q-PCR were Forward-eGFP: 5′-GTCCGCCCTGAGCAAACA-3′ and Reverse-eGFP: 5′-TCCAGCAGGACCATGTGATC-3′. Vector titers obtained ranged from 2 × 10^12^ to 9 × 10^12^ vp/ml.

### Histological procedures

Mice were sacrificed 3 weeks post-surgery by transcardiac perfusion with saline Ringer solution followed by 4% paraformaldehyde in 0.1 M phosphate buffer (PB). Brains were dissected and stored for 48 h in a cryopreservation solution containing 10% glycerin and 2% dimethylsulphoxide (DMSO) in 0.125 M PB, pH 7.4 at 4°C. Frozen serial coronal sections (40 μm thickness) were obtained using a sliding microtome and collected in cryopreservation solution in series of 10 adjacent sections.

Free-floating sections were rinsed with Tris buffer pH 7.4 (TBS) and then incubated in a blocking solution containing 1% cold fish gelatin (Sigma), 1% bovine serum albumin (BSA), and 0.05% Triton X-100 in TBS for 1 h; sections were then incubated overnight at room temperature (RT), with the appropriate primary antibodies diluted in blocking solution.

The following primary antibodies were used in double immunofluorescent stains: (1) rabbit anti-GFAP (1:400, Dako, Glostrup, Denmark; catalog number Z0334). (2) Mouse anti-GFAP 1:400, AbD Serotec, Killington, UK; catalog number 4650-0309). (3) Mouse anti-neuronal nuclear antigen (NeuN; 1:500, Millipore, Darmstadt, Germany; catalog number MAB 377). (4) Goat anti-olig2 (1:200, R&D systems, Minneapolis, MN; catalog number AF2418). (5) Rabbit anti-Iba1 (1:500, Wako, Neuss, Germany; catalog number 019-19741). After rinsing with TBS, sections were incubated with the appropriate fluorescent secondary antibodies diluted as before for 1 h. The following secondary antibodies were used (all purchased from Molecular Probes and used 1:200): Alexa Fluor® 633 donkey anti-rabbit IgG (#A21070), Alexa Fluor® 633 donkey anti-mouse IgG (#A21050); Alexa Fluor® 546 donkey anti-mouse IgG (#A10036); Alexa Fluor® 633 donkey anti-goat IgG (#A21080); Alexa Fluor® 555 donkey anti-rabbit IgG (#A31572), Alexa Fluor® 546 goat anti-rabbit (#A11010) Alexa Fluor® 546 goat anti-mouse (#A11003). Finally, sections were rinsed in PBS and mounted on SuperFrost Ultra Plus slides, dried at RT and coverslipped with Depex (VWR International). As negative control and to verify the specificity of the secondary antibodies, the same immunohistochemistry procedure was performed omitting the primary antibodies. No staining was observed. Furthermore, all antibodies used here were used in other publications (see Eng et al., 2000; Talbott et al., 2007; Gil-Perotin et al., 2009; Lalancette-Hebert et al., 2012; Seto et al., 2014; Haberl et al., 2015). Sections were inspected under a confocal laser-scanning microscope (LSM 800; Zeiss, Jena, Germany). To ensure appropriate visualization of the labeled elements and to avoid false positive results, the emission from the argon laser at 488 nm was filtered through a band pass filter of 505–530 nm and color-coded in green. The emission following excitation from the helium laser at 543 nm was filtered through a band pass filter of 560–615 nm and color-coded in red. A long-pass filter of 650 nm was used to visualize the emission from the helium laser at 633 nm and color-coded in pale blue.

As the main goal of this study was to determine the specificity of the chosen promoters in the context of AAV8-mediated gene delivery to the striatum, we focused our analysis on the transduced area only. The numbers of eGFP-positive cells infected with each vector were determined on images of eight random areas within the transduced striatum regions (i.e., containing at least one eGFP^+^ cell) per mouse using a 40x objective and ImageJ software. Percentages were calculated based on the total number of transduced cells (number of eGFP^+^NeuN^+^/total NeunN^+^, eGFP^+^GFAP^+^/total GFAP^+^, or eGFP^+^Olig2^+^/total Olig2^+^, respectively).

### Statistical analysis

The results were expressed as mean ± standard deviation (*SD*). Statistical analyses were performed using the software GraphPadPrism. To test for difference in transduction efficacy, a non-parametric one-way ANOVA with Tukey post-test was applied, except for **Figure 5** where we used Chi square analysis. All tests were considered significant if *p* < 0.05.

## Results

### *In vitro* analysis

**Figure 2:**
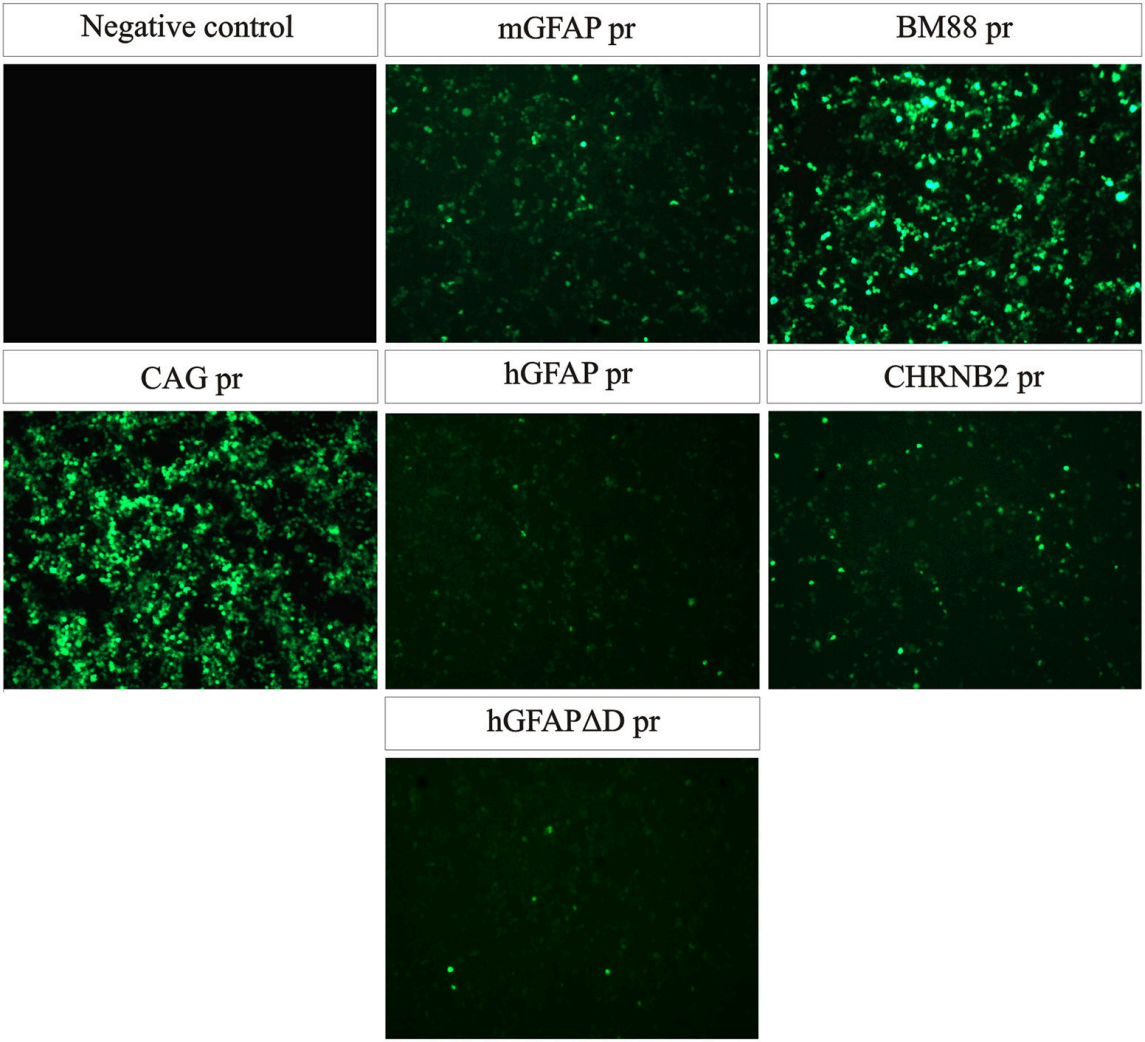
**All plasmid constructs are functional *in vitro***. HEK-293T cells were transfected with the same amount of each plasmid and 48 h later eGFP expression was analyzed. All promoters were able to drive transgene expression, the strongest one being BM88 and the weakest one hGFAP. As positive control, cells were transfected with a plasmid expressing GFP under the control of the strong and ubiquitous promoter CAG.

A total of six recombinant AAV genomes carrying an eGFP reporter gene were constructed (Figure 1; for a more detailed description of the vectors see Section Materials and Methods). In brief, five constructs carried CNS cell-specific promoters and one a ubiquitous promoter, CAG pr, which was used as control. Three promoters targeting astrocytes were tested: hGFAP pr, hGFAPΔD pr, and mGFAP pr, as well as two neuronal ones, BM88 pr and CHNRB2 pr. The expression of eGFP, driven by the different constructs, was first analyzed *in vitro* by plasmid transfection of HEK-293T cells (Figure 2). All promoters were able to drive the expression of the fluorescent protein, however, important differences in their transcriptional activity were found. Of the neuronal promoters, BM88 was stronger than CHNRB2, while the astrocyte promoter mGFAP was better than hGFAP, and the level of eGFP expression was not diminished upon deletion of the D region. Non-transfected controls were eGFP negative whereas the majority of cells transfected with plasmid containing eGFP under the control of CAG pr were strongly positive.

### Analysis of the transduction efficacy of the different vectors in mouse striatum

**Figure 3:**
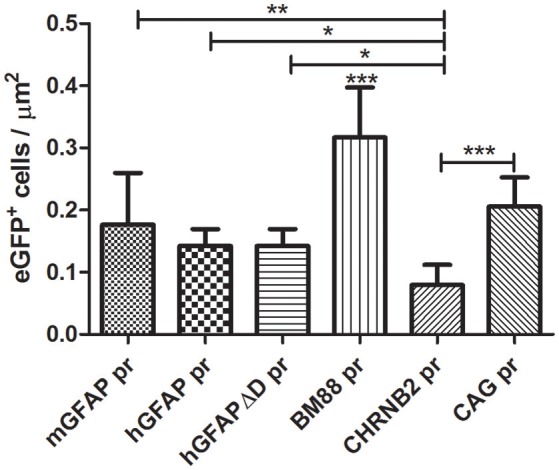
**Analysis of the transduction efficacy of the AAV8 vectors carrying different promoters**. Mice were treated with the different vectors (4 × 10^9^ vp/mouse) by stereotaxic surgery in the left striatum. Twenty-one days later mice were sacrificed and the number of eGFP-positive cells in the transduced area was quantified (mGFAP = 648 cells; hGFAP = 360 cells; hGFAPΔD = 365 cells; BM88 = 808 cells; CHRNB2 = 204 cells; CAG = 525 cells). Mean ± *SD* are shown. Differences in the number or GFP^+^ cells were statistically evaluated by One-way ANOVA. Results were considered significant when *p* < 0.05 and levels of significance are indicated as follows: ^*^p < 0.05; ^**^p < 0.01; ^***^p < 0.001.

After testing the functionality of the different plasmids *in vitro*, we produced the recombinant AAV8 vectors for *in vivo* studies. A dose of 4 × 10^9^ vp of each AAV8-eGFP vector was injected into the left striatum by stereotactic injection (*n* = 3 per group). The mice did not display any adverse reaction or behavioral changes after the intracranial surgery or during the subsequent period until sacrifice. However, no long term studies were performed to test the potential toxicity of sustained transgene expression. Three weeks after vector injection mice were euthanized and eGFP expression was analyzed in both the right and left striatum. In all groups eGFP expression was detected in the left striatum as well as along the injection tract but never in the right brain hemisphere (See Supplementary Figure 1). The number of eGFP-positive cells/μm^2^ varied depending on the promoter. The highest number of transduced cells was observed with the vector carrying the neuron-specific promoter BM88 pr (Figure 3). Transduction was significantly less efficient using the three variants of GFAP, CHNRB2, or CAG as promoter. The lowest levels of expression were consistently found with the construct containing CHRNB2 pr.

### Transduction of neurons and astrocytes by CAG-driven AAV8

**Figure 4:**
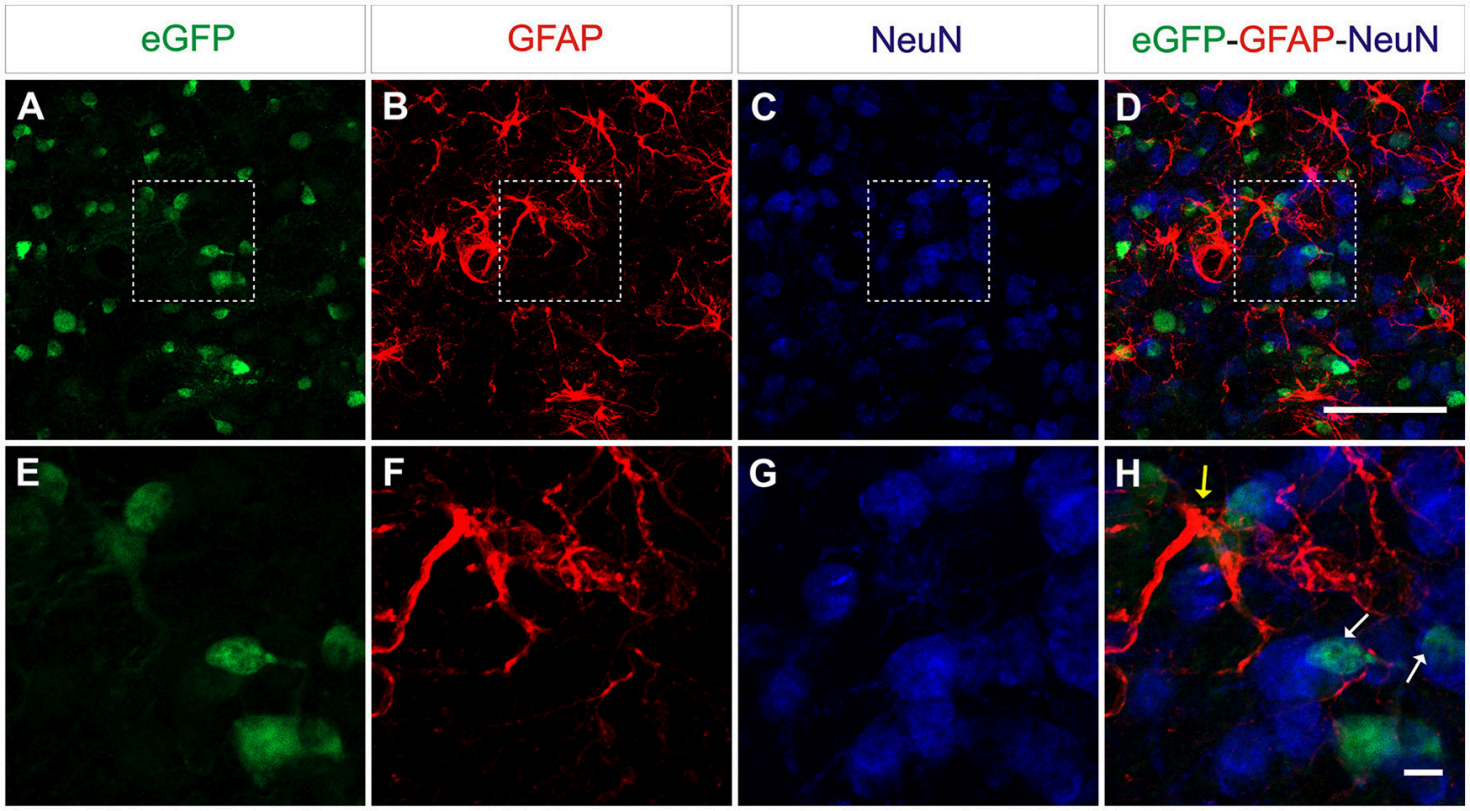
**AAV8 expressing eGFP under the control of an ubiquitous promoter transduce both neurons and astrocytes efficiently**. Adult mice were injected with 4 × 10^9^ vp/mouse and killed 3 weeks post-injection to determine eGFP^+^cells (green) in the striatum. Neurons were labeled with an anti-NeuN antibody (blue) and astrocytes were labeled with an anti-GFAP antibody (red). Arrows indicate eGFP double-positive cells (yellow = eGFP^+^/GFAP^+^, white GFP^+^/NeuN^+^). Scale bars 20 μm **(A–D)** and 5 μm **(E-H)**.

**Figure 5:**
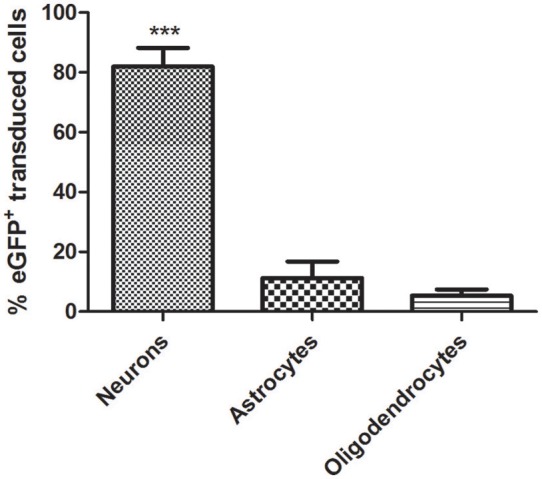
**Quantitative analysis of AAV-CAG-eGFP transduction**. Although the AAV8 vector transduces both neurons and astrocytes and CAG is a ubiquitous promoter, neuronal cells are transduced significantly better. (Neurons = 1311/1577 eGFP^+^; astrocytes = 180/1577 eGFP^+^; oligodendrocytes = 86/1577 eGFP^+^). Mean ± *SD* are shown. Analysis was restricted to the transduced area of the striatum as described in Methods. Differences in the number of eGFP^+^ cells were statistically evaluated by Chi square analysis. Results were considered significant when *p* < 0.05; ^***^p < 0.001.

CNS tissue is highly heterogeneous and consists of different cell types including neurons and glia cells. Following the delivery of eGFP expressing vectors under the control of the constitutive promoter CAG, eGFP-expressing cells with different morphologies were observed. To further identify the type(s) of cells transduced by this vector, a triple immunofluorescence stain using both anti-eGFP as well as cell-specific markers was performed. Astrocytes were identified by their expression of GFAP, whereas for neurons the pan-neuronal marker of neuronal nuclei (NeuN) was used (Figure 4). AAV8-CAG-eGFP mainly transduced neuronal cells and to a lesser extent also astrocytes and oligodendrocytes. Quantification of eGFP-expressing cells revealed that the number of eGFP-positive neuronal cells was eight-fold higher than the number of astrocytes (Figure 5). These results indicate that both neurons and astrocytes are transduced by AAV8 after stereotactic injection into the striatum, albeit with a greatly varying efficacy.

### Astrocytic transgene expression is driven by GFAP promoters

**Figure 6:**
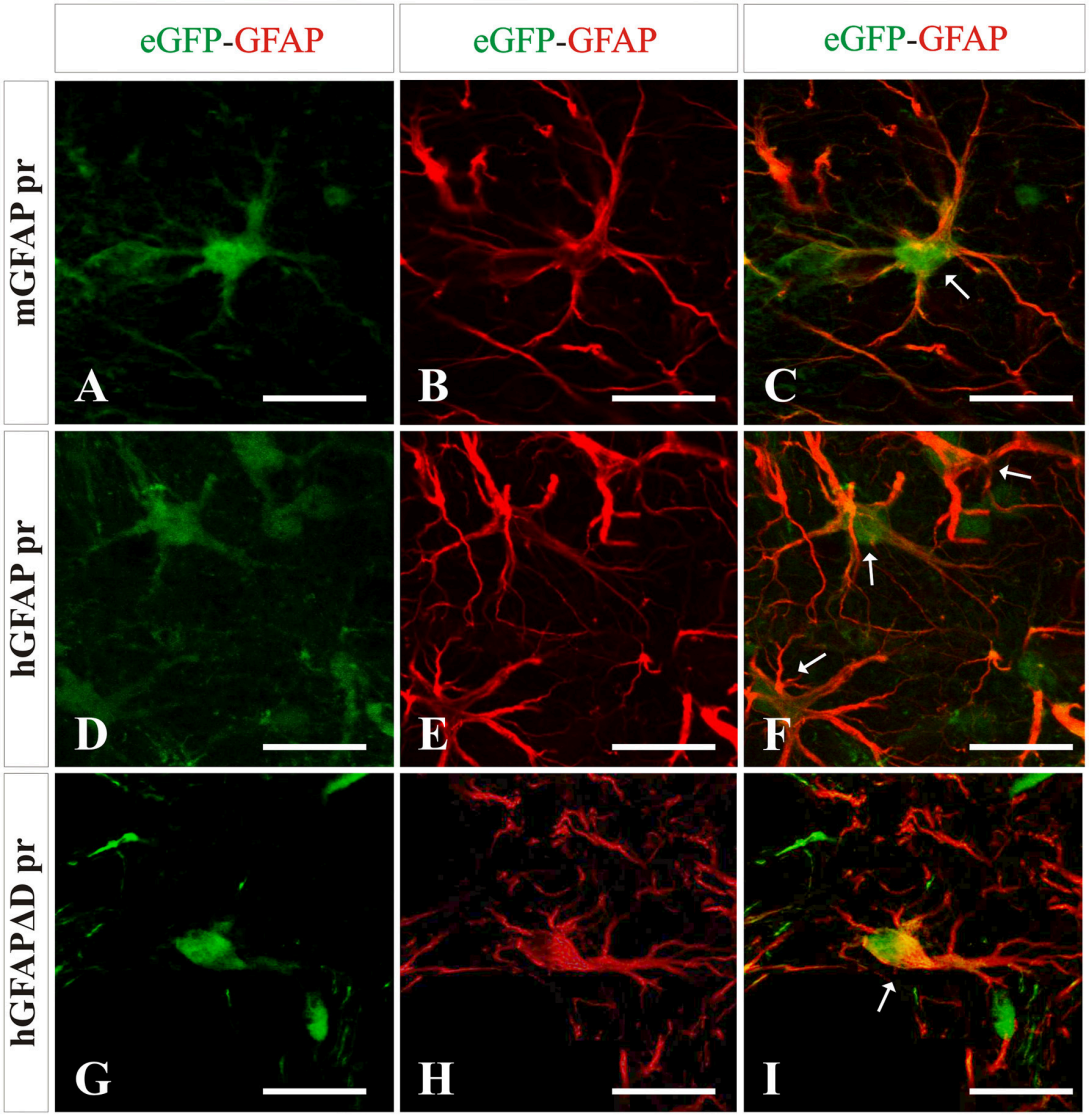
**Analysis of brain transduction by AAV8 carrying eGFP under the transcriptional control of astrocyte-specific promoters**. Mice were treated with the different vectors carrying astrocyte-specific promoters mGFAP pr **(A–C)**, hGFAP pr **(D–F)**, and hGFAPΔD pr **(G–I)** at the same dose by stereotaxic surgery in the left striatum. Twenty-one days later mice were sacrificed and the number and type of eGFP^+^ cells (green) were analyzed. Astrocytes were labeled with an anti-GFAP antibody (red). A clear co-localization of GFAP and transgene expression is indicated by arrows. Scale bars 20 μm.

**Figure 7:**
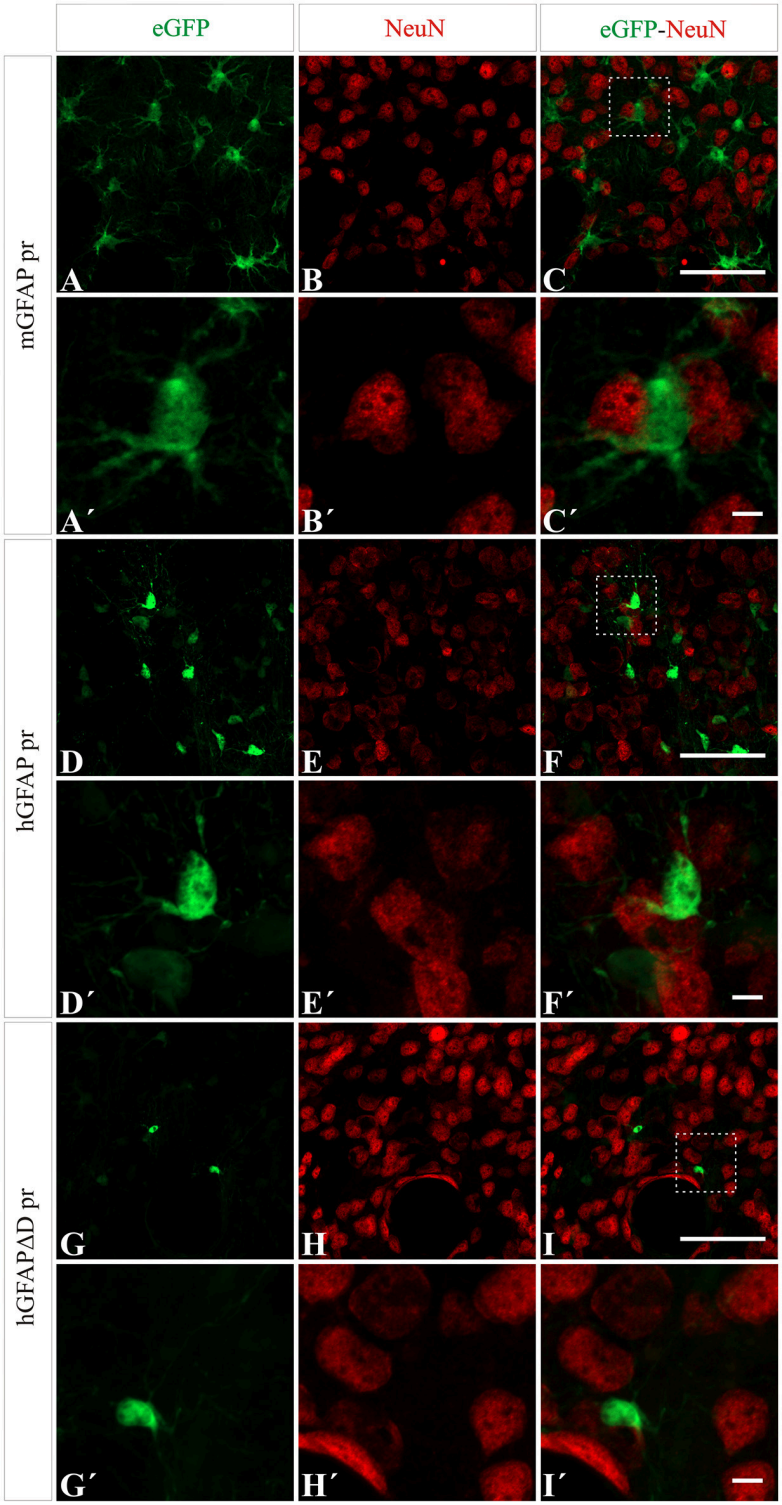
**No transgene expression in neurons is detected when using AAV8 carrying GFAP derived promoters**. NeuN expression (red) did not co-localize with eGFP expression (green) after injection with AAV8 carrying the mGFAP **(A–C)**, hGFAP **(D–F)**, and hGFAPΔD **(G–I)** promoters. Scale bars: low magnification—20 μm **(A–I)** and high magnification—5 μm **(A′–I′)**.

**Figure 8:**
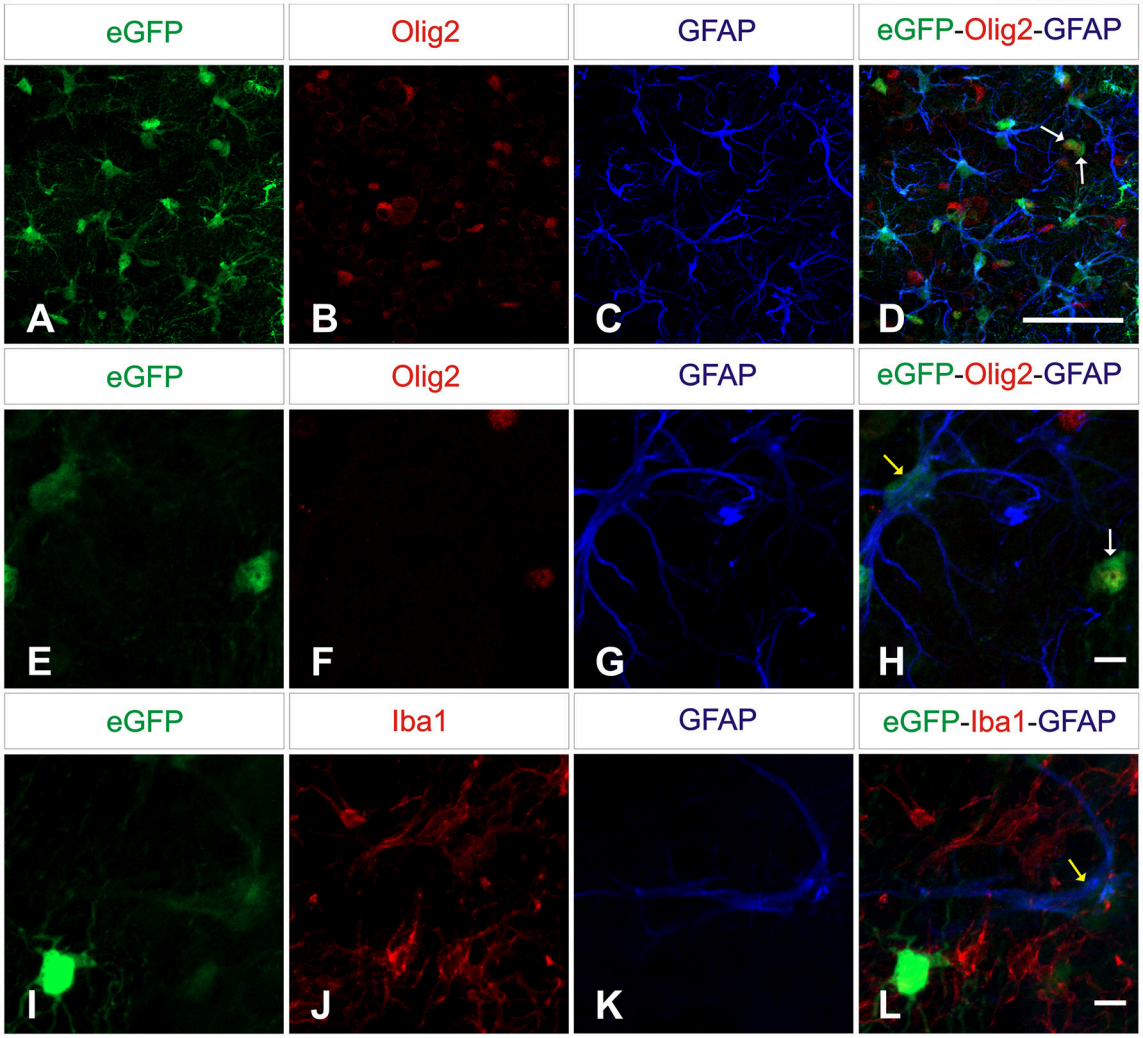
**AAV8 carrying GFAP-derived promoters drive transgene expression in oligodendrocytes**. Brain sections were labeled with anti-Olig2 (red; upper and middle panels) or anti-Iba1 (red; lower panels) and anti-GFAP (blue) to differentiate microglial, oligodendrocytes and macroglial. Olig2 immunoreactive cells **(D,H)** and astrocytes **(D,H,L)** showed co-expression of eGFP (green; arrows: yellow = GFP^+^/GFAP^+^, white = GFP^+^/Olig2^+^) while Iba1-positive cells were negative. Scale bars 20 μm **(A–D)** and 5 μm **(E–L)**.

Next we wanted to analyze the specificity of the vectors containing either neuron or astrocyte-specific promoters. In mice injected with either of the AAV-GFAPpr variants most of the eGFP-positive cells were astrocytes (Figure 6), while neurons were never found to express eGFP (Figure 7). Interestingly, a population of eGFP-positive cells lacking GFAP expression was detected. These cells were lacking the morphological features that typically characterize astrocytes and their small size and morphology were consistent with an oligodendroglial phenotype. In an attempt to properly identify the exact nature of these cells, we labeled brain sections with anti-eGFP, anti-Olig 2, an oligodendroglial marker, and anti-Ibal, a microglial marker. As shown in Figure 8, eGFP co-localized with Olig2-expressing cells but not with Ibal.

**Figure 9:**
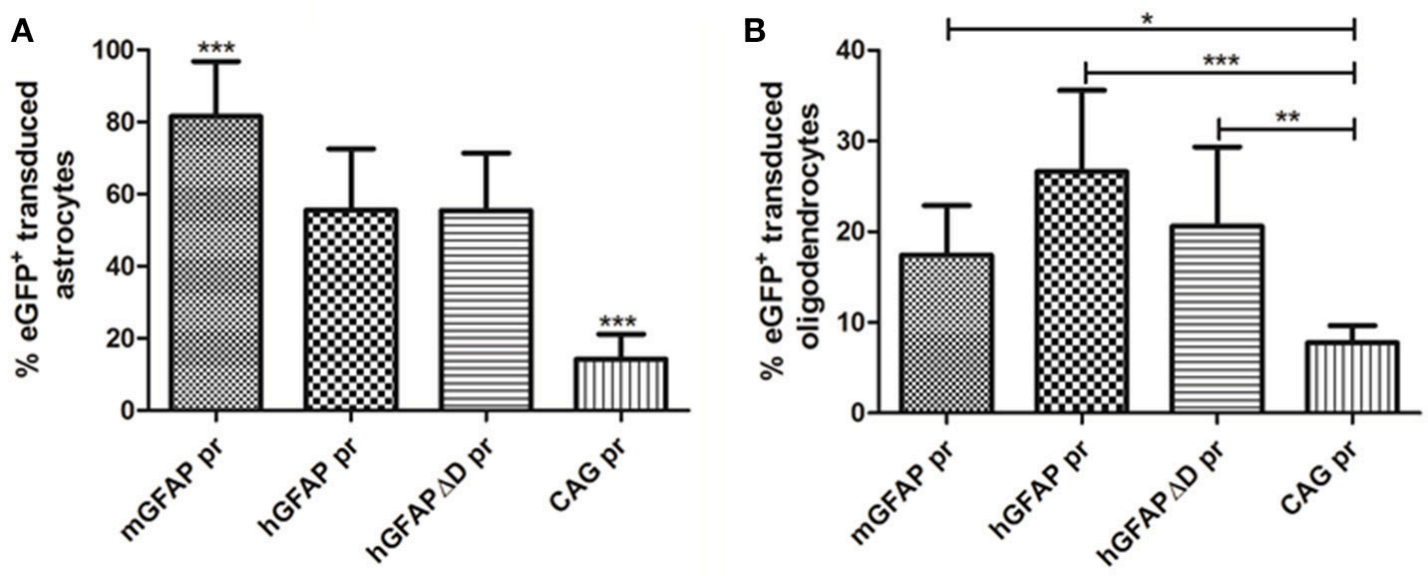
**Quantification of the percentage of eGFP+ astrocytes and oligodendrocytes transduced by AAV8 carrying GFAP-derived promoters. (A)** The vast majority of transgene expressing cells after AAV-GFAP-eGFP injection were astrocytes. The percentage of eGFP^+^ astrocytes/total GFAP^+^ cells is plotted (mGFAP = 550/648 cells; hGFAP = 206/360 cells; hGFAPΔD = 98/186 cells; CAG = 28/224 cells). **(B)** A smaller fraction of oligodendrocytes was eGFP^+^ (mGFAP = 22/131 cells; hGFAP = 41/157 cells; hGFAPΔD = 40/186 cells; CAG = 16/224 cells). Mean ± *SD* are shown. Counting was restricted to the transduced area of the striatum as described in Methods. Differences in the number of eGFP^+^ cells were statistically evaluated by One-way ANOVA. Results were considered *p* < 0.05 and levels of significance are indicated as follows: ^*^p < 0.05; ^**^p < 0.01; ^***^p < 0.001.

Mice injected with the vector carrying the mGFAP promoter had the highest levels of transduction. A strong fluorescence was observed in cell bodies throughout the dorsal area of the striatum. Furthermore, the number of positive cells was similar in the groups injected with either hGFAP or hGFAPΔD, indicating that the D element is dispensable for the transcriptional activity of the promoter (no significant difference was observed between these two promoters; Figure 9A). This is in line with what was suggested by our *in vitro* results. Three weeks after viral injection, 81.5% of astrocytes in AAV8-mGFAP recipients were eGFP positive. With 55.5% of positive cells in AAV8-hGFAP- and AAV8-hGFAPΔD-injected mice, respectively, these promoters were less efficient (Figure 9A). Moreover, co-localization of reporter gene expression and the oligodendroglia marker Olig2 in the AAV8-mGFAP group was the lowest with 17.4% (Figure 9B). With the other variants 20% (AAV8-hGFAPΔD) and 26.6% (AAV8-hGFAP), respectively, stained positive for both eGFP and Olig2. These results indicate that the mGFAP promoter works best for astrocyte-specific transgene expression.

### CHB2RN and BM88 promoters induce neuronal transgene expression

**Figure 10:**
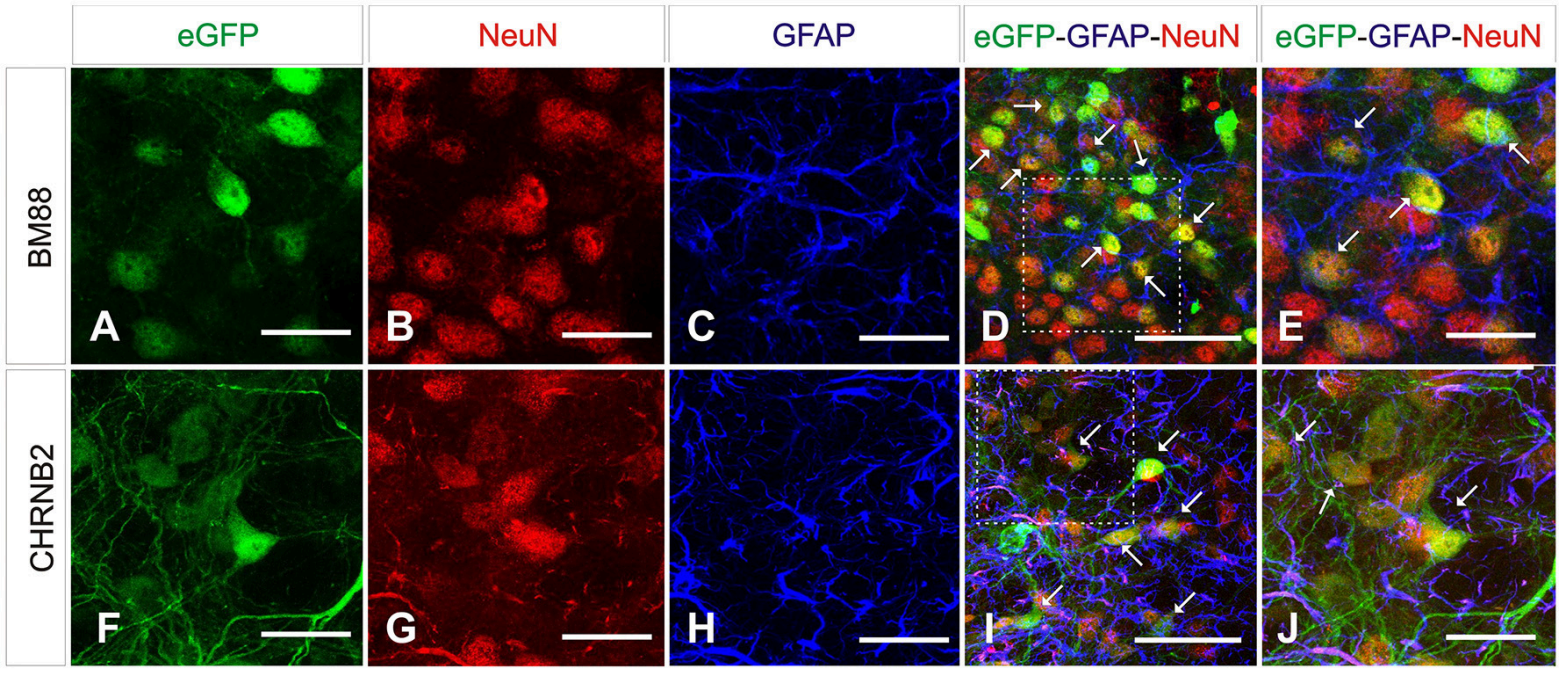
**Analysis of brain transduction by AAV8 carrying eGFP under the transcriptional control of neuronal promoters**. Mice were treated with AAV8 carrying the neuronal promoters BM88 **(A–E)**, CHB2RN **(F–J)** and the eGFP reporter gene as before. Twenty-one days later mice were sacrificed and the number and type of eGFP^+^ cells was analyzed. NeuN^+^ cells (red) showed a clear co-expression of eGFP (green; arrows), while astrocytes expressing GFAP (blue) did not express eGFP (green). We also observed NeuN^+^ neurons that were not expressing eGFP. Scale bars: D, I 20 μm. All others 50 μm.

**Figure 11:**
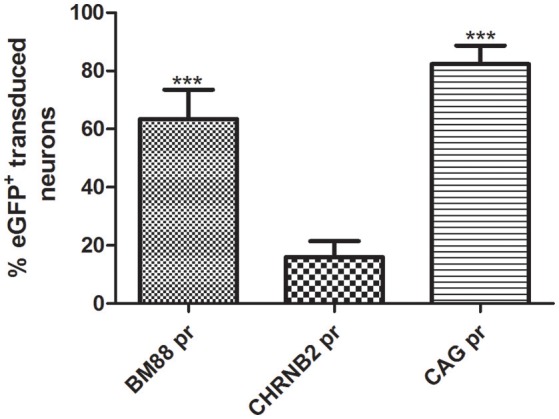
**Quantification of the percentage of neuronal cells expressing eGFP after AAV8 injection carrying BM88 and CHB2RN promoters**. A high percentage of neuronal cells are transduced using the minimal promoter BM88, higher neuronal transduction efficiency was obtained in comparison to CHBRN and CAG promoters. The percentage of eGFP^+^neurons/total NeuN^+^ cells is shown (BM88 = 808/1302 cells; CHRNB2 = 204/1250 cells; CAG = 473/525 cells). Mean ± *SD* are plotted. Counting was restricted to the transduced area of the striatum as described in Methods and differences in the number or eGFP^+^ cells were statistically evaluated by One-way ANOVA. The significance level was set to ^***^p < 0.001.

Selective neuronal transduction was observed in mice having received the vector in which eGFP had been placed under control of the CHNRB2 or BM88 promoters (Figure 10). Widespread expression of the transgene was observed throughout the dorsal region of the striatum. At higher magnification specific eGFP expression was identified within NeuN-positive cells (Figure 10), suggesting that eGFP-expressing cells accounted for a neuronal phenotype. This is supported by the fact that co-localization of eGFP and GFAP was never found. The percentage of NeuN-positive cells co-expressing eGFP was significantly higher in animals injected with the BM88 vector (63.4%) than in those injected with the CHNRB2 promoter (15.9%), thus identifying the former as the more useful one for expressing transgenes in neuronal cells (Figure 11).

## Discussion

Here, we described the development and transduction efficacy of AAV-based gene delivery vectors for cell-specific transgene expression in the CNS.

In order to achieve a successful delivery and expression of the therapeutic gene, selection of both delivery vehicle and an optimal expression cassette is essential. As vehicle we chose AAV serotype 8 as it was previously shown to more efficiently transduce cells of the CNS than other serotypes (Aschauer et al., 2013) and to also infect a larger area than the best described and most commonly used serotype 2 (Watakabe et al., 2015). The packaging capacity of serotypes 2 and 8 (or any other AAV serotype) does not differ and expression cassettes are generally based on AAV2. However, having by far the smallest packaging capacity amongst the viruses used for gene therapy (4.4–4.7 kb), a need for a reduction in promoter elements is obvious in order to allow the packaging of larger genes or multiple genes. Accordingly we here focused on the identification of small or minimal cell-specific promoters for CNS applications.

The functionality of the newly generated plasmids was first characterized *in vitro* by transfection into HEK-293T cell lines. Although derived from human embryonic kidney cells, previous reports demonstrated that this cell line expresses significant amounts of proteins found in the CNS, such as neurofibroblast subunits and α-internexin (Shaw et al., 2002). HEK-293T cells also express different neuronal receptors and electrophysiological studies have shown the presence of endogenous voltage-activated ion currents (Shaw et al., 2002), which supports the use of this cell line for testing the performance of the CNS-specific promoters. We indeed observed that all the “cell-specific” promoters were able to drive the expression of the reporter gene. That the transduction of HEK293T cells with AAV carrying the neuron-specific promoter BM88 was significantly more efficient than with all other promoters—including the ubiquitous promoter CAG—is easily explained by the fact that the observed expression pattern of CNS-specific proteins in HEK-293T cells is similar to that of a typical early differentiating neurons or neuronal stem cells.

AAV8 has been described to be highly efficient in driving eGFP expression in astrocytes and neurons in the striatum (Taymans et al., 2007) and we first tested the transduction efficiency and efficacy of an eGFP-expressing AAV8 vector under the control of the ubiquitous and highly potent promoter CAG. After stereotactic delivery into the mouse striatum both astrocytes and neurons were found to be transduced with our vector, the latter even more efficiently.

Interestingly, previous experiments using an AAV8 with a similar construct showed a better performance for astrocyte- rather than neuronal transduction in the striatum (Aschauer et al., 2013). Using a ubiquitous promotor in macaques resulted in transduction of certain neuronal cell subtypes but not glia (Masamizu et al., 2010). The main difference between our study and that of Aschauer et al. is that they purified the AAV vector by CsCl density gradient centrifugation, whereas we here performed an iodixanol gradient. Differences in transduction-efficacy and -specificity were shown previously to not only depend on the AAV capsid serotype but to also be related to the production and purification methods (Ayuso et al., 2010).

Astrocytes are the most abundant cell type in the vertebrate CNS and hence involved in many degenerative diseases. We therefore characterized reduced forms of the human and murine GFAP promoters, which mainly drive transgene expression in astrocytes. Importantly, because GFAP is not expressed in neurons, these promoters cannot drive neuronal expression of the transgene. The reduced form of the hGFAP promoter used in this study was previously characterized *in vitro* by Lee et al. (2008). We further reduced its size by removing the D element located at the 3′ end of the promoter sequence. Deletion of the D element was reported to severely reduce transcription (Besnard et al., 1991; Lee et al., 2008), however, our *in vivo* data indicates that this element is not essential for the transcriptional activity of the promoter in astrocytes. In addition we tested the transcriptional activity of the reduced version of the mGFAP promoter, which was designed using the reduced version of the hGFAP (Lee et al., 2008) as a model. The murine version of the promoter was more active and specific than the human one.

Co-localization of eGFP/GFAP was observed in the striatum of all mice injected with either of the three GFAP promoters, demonstrating that they all efficiently transduce astrocytes. We also found a proportion of eGFP-expressing GFAP-negative cells that were subsequently identified as oligodendrocytes. Previous work reported weak neuronal expression using GFAP promoter, but oligodendrocyte expression has not been reported (Lee et al., 2008). Currently we do not have an explanation for this finding. Even though expression of hGFAP (but not mGFAP) was shown in an oligodendrocyte precursor cell (Casper and McCarthy, 2006), the infection and subsequent maturation of these precursors can be excluded as the same precursor can give rise to neuronal cells and eGFP expression was not seen in either neurons or microglial cells. Interestingly, while the transduction efficacy of astrocytes was significantly better with the murine GFAP promoter, transduction of oligodendrocytes did not significantly differ between the three GFAP variants.

While we observed a degree of axonal transport of both AAVs, more experiments need to be performed to determine whether this was retrograde or anterograde. In mice, vector transport along astrocytes has been described in previous studies and was found to be serotype dependent (anterograde: AAV2, Salegio et al., 2012; retrograde: AAV5, Aschauer et al., 2013). Other studies done in marmoset and macaque using AAV8 revealed preferential retrograde transport of this serotype (Masamizu et al., 2011). Future experiments will allow us to confirm the direction of the axonal transport. Another of our future aims is to use GFAP promoters for the astrocyte-selective expression of genes coding for several transcription factors with the ultimate goal of conducting *in vivo*-reprogramming of these astrocytes into neurons.

For neuron-specific expression we used two very small promoters with different transcriptional potencies, BM88 and CHNB2. The minimal promoter derived from the neural protein BM88 had a stronger transcriptional activity and in the transduced area up to 63% of striatal neurons were eGFP^+^. This can be explained by the expression pattern of BM88: it is not only widely expressed in proliferating neuronal precursors, but also at an even higher level in their post-mitotic neuronal progeny in the developing as well as in the adult brain (Koutmani et al., 2004). In contrast, the minimal CHRNB2 promoter was also neuron specific but transgene expression was a lot lower and found in only <20% of NeuN^+^ cells. This result was somehow surprising since the CHNB2 pr controls the expression of the nicotinic receptor β-subunit, which is expressed in the majority of neurons in the brain. However, it can possibly be explained by the fact that we are using a reduced version of the promoter, whose activity is significantly lower than that of the original promoter (Bessis et al., 1995). Thus we are most likely unable to detect eGFP expression in all the cells that have been transduced. Moreover, the distribution of the nicotinic acetylcholine receptors subtypes expressed in the CNS will have a direct effect on the promoter activity (Gotti et al., 2006). Additional studies will be needed to better characterize of the type of neurons transduced by either promoter. The small size of these promoters allows the expression of larger genes or more than one gene in neurons. Diseases caused by the deficiency of large genes are not uncommon among the spectrum of neurological disorders, such as for instance in autism spectrum disorders, intellectual disability, or Dravet syndrome, in which mutations in several genes are involved. Thus, development of vectors allowing the insertion of multiple genes would be of paramount importance for the adequate development of gene therapy approaches when dealing with these diseases.

In conclusion, we have developed and characterized AAV-vectors with a relatively large cloning capacity for the cell-specific delivery of therapeutic genes to the CNS. Albeit yet needing further characterization these cell-specific AAVs represent promising tools with a great potential use for the development of gene therapy approaches for neurodegenerative disorders.

## Author contributions

Study concept and design: DP, LV, and GG; Acquisition, analysis, and interpretation of data: DP, DS, LV, EL, and ID; Drafting of the manuscript: DS and DP; Critical revision of the manuscript: MH, JL, and GG; Statistical analysis: DP and DS; Obtained funding: JL and GG; Study supervision: GG. Technical assistance: AV; Critical revision of the manuscript: AR.

## Funding

Supported by FP7-PEOPLE-2011-IAPP—Marie Curie Action: “Industry-Academia Partnerships and Pathways” (ref. 286071 “Brainvectors”) and ERC Advanced grants (CoEN-Pathfinder Phase II Call; ref. 340527 “Repropark”). Diego Pignataro is partially supported by a Jon Zarandona donation.

### Conflict of interest statement

The authors declare that the research was conducted in the absence of any commercial or financial relationships that could be construed as a potential conflict of interest.

## Supplementary material

Supplementary Figure 1 **Striatal transduction area for each viral vectors**. Representative images showing the different patterns of viral spread in mice striatum. Scale bar: 2000 μm.
