# Supplementary figures and images for: Adeno-Associated Viral Vectors Serotype 8 for Cell-Specific Delivery of Therapeutic Genes in the Central Nervous System
Source: Front Neuroanat. 2017 Feb 10;11:2. doi: 10.3389/fnana.2017.00002 (PMC5301009; doi:10.3389/fnana.2017.00002)

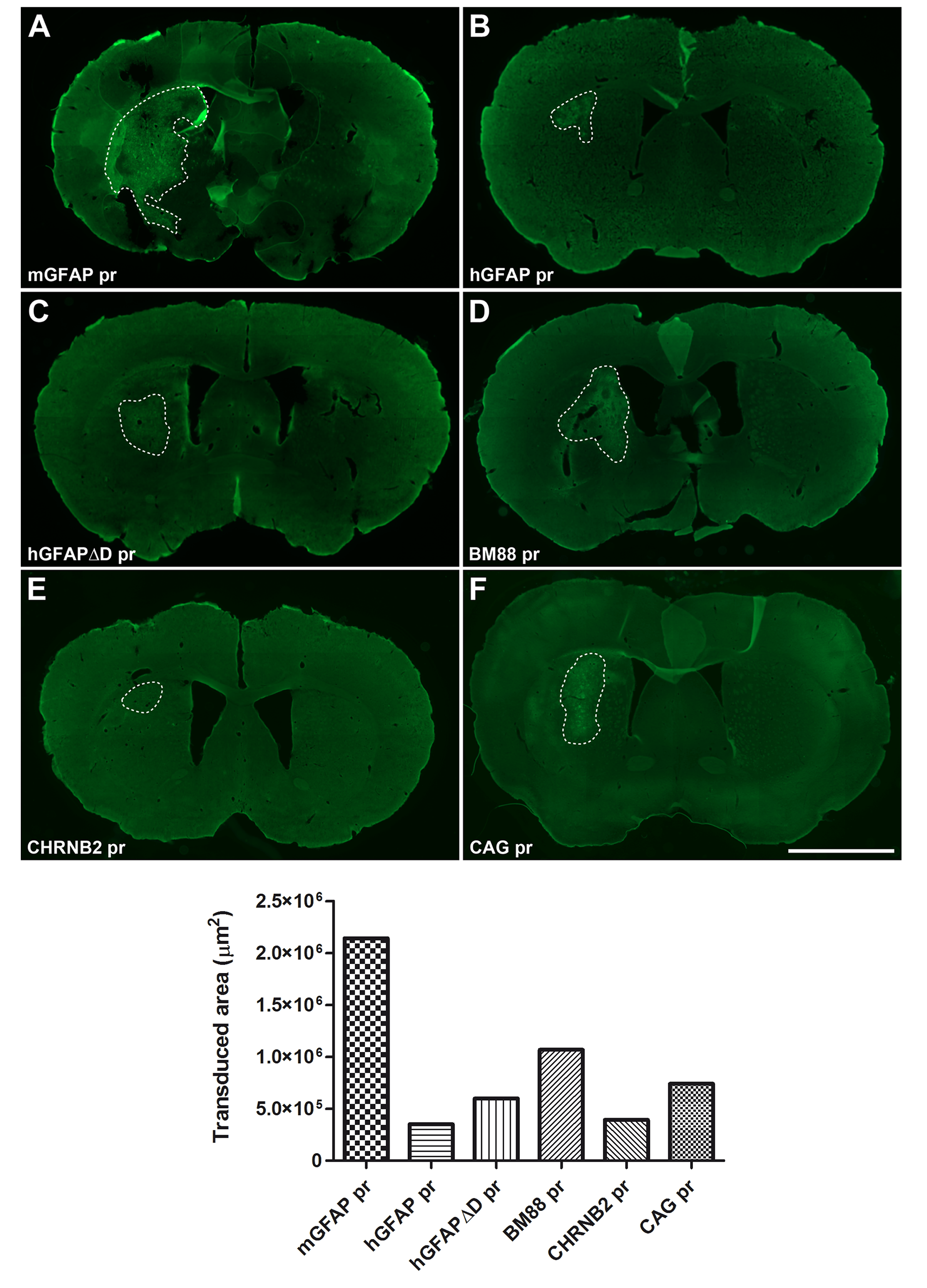

Supplement: Supplementary Figure 1 — Striatal transduction area for each viral vectors. Representative images showing the different patterns of viral spread in mice striatum. Scale bar: 2000 μm. [file Image1.tif]
